# A rare case of nonresterilized reinforced ETT obstruction caused by a structural defect: A case report

**DOI:** 10.1097/MD.0000000000008886

**Published:** 2017-12-01

**Authors:** Sang Ho Kim, A Yeon Park, Ho Bum Cho, Jae Hwa Yoo, Sun Young Park, Ji Won Chung, Mun Gyu Kim

**Affiliations:** Department of Anesthesiology and Pain Medicine, Soonchunhyang University Hospital Seoul, Hannam-dong, Yongsan-gu, Seoul, Korea.

**Keywords:** endotracheal tube, peak inspiratory pressure

## Abstract

**Rationale:**

Various factors can cause ventilatory failure after endotracheal tube (ETT) intubation, which is associated with increased patient morbidity and mortality.

**Patient concerns:**

A 76-year-old woman who was diagnosed with a hemopericardium and suspicion of a major-vessel injury due to dislocation of the clavicular fracture fixation screw.

**Diagnosis:**

Non-resterilized reinforced ETT obstruction caused by a structural defect.

**Intervention:**

Endotracheal tube was exchanged.

**Outcomes:**

The ventilator profile showed rapid improvement.

**Lessons:**

Anesthesiologists should consider that a non-resterilized reinforced ETT may be defective. An ETT defect can cause high PIP and ETT obstruction without kinking or foreign materials.

## 1 Introduction

Ventilatory failure during general anesthesia is a principal cause of morbidity and mortality; therefore, there is a need to confirm airway patency. Among the many airway maintenance methods, endotracheal tube (ETT) intubation is the most general and safe method for general anesthesia. However, ETT intubation does not guarantee airway patency, and the ETT itself can become a source of airway obstruction.^[1]^ Furthermore, it is difficult to detect an ETT defect quickly, and the results of the defect can be misjudged as being other clinical conditions.

Here, we present a rare case of nonresterilized reinforced ETT obstruction caused by a structural defect; the hemodynamically unstable patient was undergoing emergency explorative sternotomy for hemopericardium.

## 2 Case report

A 76-year-old woman (weight, 52 kg and height, 158 cm) classified as American Society of Anesthesiologists (ASA) physical status IV E, was admitted due to sudden chest discomfort and dyspnea. She had hypertension, hyperlipidemia, and a history of surgical fixation of the clavicle with a screw for a clavicular fracture 5 days previously. She underwent chest radiography and chest computed tomography (CT) in the emergency room (ER) and was diagnosed with a hemopericardium and suspicion of a major-vessel injury due to dislocation of the clavicular fracture fixation screw. Her hemoglobin level was 5.9 mg/dL, suggestive of ongoing bleeding. An emergency operation was planned, and 2 pints of packed red blood cells were immediately transfused. Her mental status was alert with spontaneous ventilation. Vital signs were blood pressure of 174/89 mm Hg, heart rate of 72/min, body temperature of 36.2°C, and respiratory rate (RR) of 36. Oxygen saturation was 90% under 3 L/min oxygen through a nasal cannula. Breathing sounds in both lung fields were clear without crackling or rhonchi.

On arrival in the operating theater without premedication, standard monitoring devices including electrocardiography, pulse oximetry, and an oscillometric noninvasive blood-pressure cuff were applied. After a few minutes of preoxygenation, general anesthesia was induced using 10 mg intravenous etomidate (0.2 mg/kg), 50 μg remifentanil (1 μg/kg), and 50 mg rocuronium (1 mg/kg) for neuromuscular blockade. Tracheal intubation was performed with a 7.0-mm I.D. reinforced nonresterilized ETT (Sheridan; Teleflex Medical, Morrisville, NC) using a McGrath videoscope (Aircraft Medical Ltd, Edinburgh, UK). The insertion depth was 22 cm from the upper incisors. Anesthesia was maintained using oxygen, medical air, 1 minimum alveolar concentration (MAC) of sevoflurane, and continuous infusion of remifentanil.

After intubation, both lung fields were auscultated using a stethoscope; there were no signs of bronchospasm, with clear lung sounds, no wheezing, and normal capnography. The ventilator was set in volume-guaranteed, pressure-controlled mode at a tidal volume of 250 mL. However, the ventilator failed to reach the intended tidal volume of 250 mL and achieved a tidal volume of only 170 mL, due to limitation in peak inspiratory pressure (PIP) of 35 cmH_2_O. We considered the high PIP to be caused by the abundant pericardial hematoma burden increasing intrathoracic pressure. We used the recruitment maneuver and applied positive end expiratory pressure (PEEP) with low tidal volume and high RR, instead of increasing inspiration pressure to maintain hemodynamic stability. An indwelling right radial artery cannula and a right internal jugular central venous catheter were placed for monitoring, blood sampling, and fluid resuscitation.

**Figure 1:**
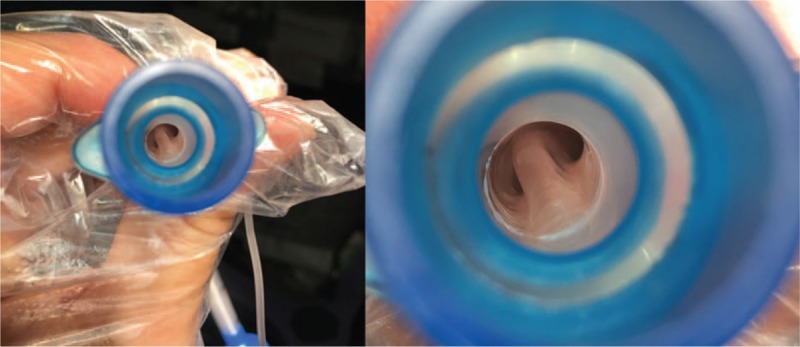
Dissection of the internal wall of a reinforced endotracheal tube (ETT).

At that time, the patient's vital signs deteriorated, with ongoing bleeding; therefore, an immediate surgical procedure became necessary. We maintained the tidal volume setting at 160 mL, RR of 16/min, and PEEP of 5 cmH_2_O and the operation began. The patient began to become desaturated after massive bleeding from the sternotomy, despite delivery of a 1.0 fraction of inspired oxygen. The oxygen saturation measurement was not reliable due to low perfusion, and the arterial line did not work appropriately for blood pressure monitoring and blood sampling. An arterial blood gas analysis (ABGA) was required to assess the adequacy of ventilation and blood transfusion; however, access to the sampling site was limited due to positioning and the ongoing procedure. Therefore, we had to wait until the main surgical procedure had been completed. After the innominate artery laceration had been found and repaired, the right femoral artery was cannulated and blood sampling was performed. The ABGA showed severe respiratory acidosis (pH 7.22, partial pressure of carbon dioxide 72.3 Torr, and partial pressure of oxygen 61 Torr). Lung compression was released once the hematoma was removed, but PIP remained higher and tidal volume lower than expected. The possibility of an ETT obstruction arose and was carefully investigated. After the recruitment maneuver, we could not advance the tracheal suction tip through the ETT, and luminal narrowing of the ETT was detected (Fig. 1).

**Figure 2:**
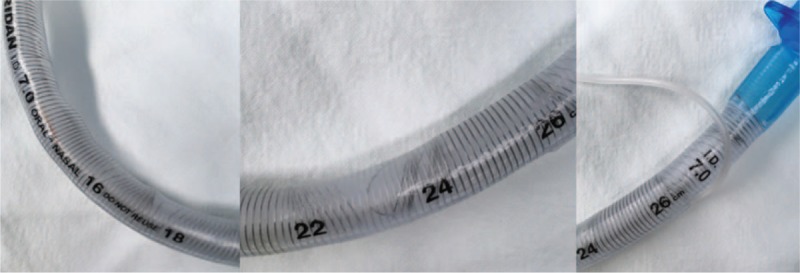
Distortion of the metal frame is evident between depth marks 16 and 18, 22 and 24, and over 26.

The tube was immediately exchanged, and the ventilator profile showed rapid improvement. The lungs ventilated with a tidal volume of 420 mL and peak airway pressure was 19 cmH_2_O with the new ETT. In addition, the ABGA results showed that the respiratory acidosis had been corrected. Almost the full length of the ETT that was removed was dissected and the metal frame was found to be distorted (Fig. 2). We assume that the faulty tube caused the increased PIP.

## 3 Discussion

ETT intubation is an essential element of general anesthesia and is needed for accurate testing to establish a secure airway. High PIP after ETT intubation can be caused by various factors such as respiratory disease and position, operation type, and problems with the ventilator or airway equipment.^[1]^ Among possible problems with airway equipment, the ETT can become obstructed by mucus, blood, or kinking. Rarely reported cases such as dissection of the internal wall, herniation of the cuff, or detachment, can also obstruct the ETT.^[2]^ Thus, accurate ETT intubation is not enough to guarantee a patent airway, and the ETT itself may become a source of airway obstruction.^[1]^ Ventilatory failure from an airway obstruction can lead to a life-threatening event; therefore, it is important to check the possible causes of ventilator failure in advance. However, such confirmation can easily be overlooked, particularly in emergency cases.

Several previous cases have reported a reinforced ETT obstruction. In most of these cases, a tube defect occurred after reusing a sterilized tube.^[1,3,4]^ Other cases of introduced nonresterilized ETT obstructions can be explained by a bleb or air formation during manufacturing, which expanded after nitrous oxide exposure.^[5,6]^ Another case report detailed an entire dissection of the inner wall of a reinforced ETT because of an aggressive stylet.^[7]^ The presumed etiology of our case was a tube-manufacturing malformation or stylet aggression of the inner wall.

During the procedure, we did not consider the possibility of a tube defect. First, we considered that the massive hematoma could increase intrathoracic pressure and PIP.^[8]^ Moreover, PIP did not increase immediately after intubation as usually occurs with an ETT defect. Therefore, the patient's high PIP was considered due to the hemopericardium. Moreover, there was no unusual resistance when the stylet was inserted and withdrawn at the moment of intubation. ETT dissection by an aggressive stylet was therefore not considered. Finally, there was neither kinking of the ETT nor change in the appearance of the ETT.

Exchanging the ETT was the solution in this case and it was not difficult or complicated. However, this situation could have worsened if ventilatory failure had been misjudged as a lung problem. Standardized checklists are available for similar conditions. The checklist confirms that critical steps are not missed and can be used in multiple situations, including on the ward, in the intensive care unit, in the operating room, and particularly during emergencies.^[9]^ In our case, airway equipment was checked by anesthetic nurses who usually check only for ETT cuff leaks and the external shape of the tube. If the anesthetic staff had checked the airway equipment more carefully, the distorted metallic frame might have been detected before intubation.

In conclusion, anesthesiologists should consider the possibility that a nonresterilized reinforced ETT may be defective. ETT defects can cause high PIP and an ETT obstruction without kinking or foreign materials. Additional careful confirmation of the checklist is an important and useful step in emergency situations.
